# Antidepressant prescribing trends for adult patients in the UK and Ireland during the COVID-19 pandemic: systematic review

**DOI:** 10.1192/bjo.2026.10990

**Published:** 2026-03-02

**Authors:** Meghann Jones, Eva M. Krockow, Samuel J. Tromans, Elizabeta B. Mukaetova-Ladinska

**Affiliations:** School of Psychology and Vision Sciences, University of Leicester, UK; Adult Learning Disability Services, Leicestershire Partnership NHS Trust, Leicester, UK; SAPPHIRE Group, Division of Public Health and Epidemiology, University of Leicester, UK; The Evington Centre, Leicestershire Partnership NHS Trust, Leicester, UK

**Keywords:** Systematic review, psychopharmacology, antidepressants, COVID-19, UK

## Abstract

**Background:**

Recent decades have seen a steady increase in antidepressant prescribing, but little is known about prescribing trends during and following the COVID-19 pandemic.

**Aims:**

This preregistered systematic review, following Preferred Reporting Items for Systematic reviews and Meta-Analyses guidelines, aimed to investigate antidepressant prescribing trends for adults in the UK and Republic of Ireland during and after the pandemic. It also compared prescriptions by drug and location.

**Method:**

We searched six databases: APA PsycInfo, CINAHL, MEDLINE, Scopus, medRxiv and Preprints.org. The review included primary research articles reporting trends in antidepressant prescriptions, including at least one time point after March 2020 in the UK and Republic of Ireland. This review has been preregistered on PROSPERO (ID: CRD42024498503).

**Results:**

We identified 7,320 studies, of which ten met the search criteria for the review. Studies were grouped on the basis of time period (2020: *n* = 5; 2021: *n* = 3; 2022: *n* = 2), location (England, Scotland, Northern Ireland, Republic of Ireland, UK) and drug type (serotonin–noradrenaline reuptake inhibitors, selective serotonin reuptake inhibitors, tricyclics, and others (e.g. monoamine oxidase inhibitors)). Most studies (eight of ten) demonstrated increased antidepressant prescribing over time. Two studies highlighted a decrease between March and May 2020. Demographic variables reflected higher rates of prescribing for women, and the modal group receiving antidepressants comprised middle-aged adults.

**Conclusions:**

The commonly reported increase in antidepressant prescribing corroborates pre-pandemic trends and may suggest further, increased demands for mental health support to meet the unique challenges of the pandemic. Future research is required to evaluate the appropriateness of treatment decisions and to explore psychosocial factors that influence individual prescribing choices.

Antidepressant prescribing has faced considerable scrutiny over the past few decades owing to a documented increase in prescriptions in the UK and other industrialised nations.^1,2^ Items prescribed in English primary care showed an increase of 52.5 million antidepressants between 1998 and 2018.^3^ Likewise, in the 5 years before the pandemic, the top 25 antidepressants demonstrated a 25% increase in the number of prescription items prescribed.^4^ This was also evident in comparisons across drug types; for instance, the number of adults being issued with at least one prescription for antidepressants between 2015 and 2018 (17%, 7.3 million prescriptions) was 4% higher than that for the next most prevalent drug class assessed (opioid pain medications; 5.6 million prescriptions).^5^ The nature of antidepressant prescriptions also showed notable changes during this period, as prescription durations and rates of repeat prescriptions increased.^6^ As a result of this continual rise, concerns have been raised around the appropriateness of antidepressant use and potential overtreatment.^2^

## The risks of overtreatment

Increasing prescribing trends for antidepressants could be an indicator of overtreatment. This refers to the prescribing of medicine despite it having a low probability of providing patient benefit and risking inciting harm, e.g. through side-effects^7^ and withdrawal effects.^6^ Overmedication has also been recognised as an especially large risk for individuals with learning disabilities and autistic people.^8^ The practice of overprescribing can result in wasted money and resources, consequently exacerbating current financial hardships faced in public healthcare. Attempts have been made to quantify the incidence of overprescribing and underprescribing of antidepressants. For instance, Davidson et al^9^ assessed adult primary care patient prescription data in Australia compared with national guidelines. They found potential undertreatment (not receiving or taking prescriptions when meeting guidelines) in 41% of patients, whereas possible overtreatment was indicated for approximately 33%. In England, for antidepressants, the yearly cost of unnecessary prescribing has been estimated to be £37.3–45.7 million, accounting for 7.6–7.9% of antidepressants prescribed.^10^ Understanding prescribing trends is a crucial step towards addressing these concerns around overtreatment. A particular period of concern is the COVID-19 pandemic, owing to its challenges to all healthcare provision. This period can also be considered pertinent for antidepressant prescribing because of noted neuropsychiatric symptoms such as depression and anxiety occurring among individuals following contraction of COVID-19.^11^ Yet, antidepressant prescribing trends during and following the pandemic have remained largely unexplored.

## International prescribing of antidepressants during the pandemic

Outside the UK and Republic of Ireland, researchers have begun to explore pandemic and post-pandemic prescribing. A study of US-based college students noted a rise in antidepressants comparing data across the 6-year period from 2015 to 2021, with this rise being most prevalent in the 2020 and 2021 pandemic period.^12^ Researchers compared the findings with expectations for prescribing following natural disasters, which typically reflect increased prescribing rates but a subsequent return to baseline levels. However, the study concluded before the end of the pandemic and left ambiguity around longer-term outcomes. This raised questions around whether there would be an eventual return to prior levels of prescribing or whether a new baseline of normalcy for rates of antidepressant prescribing would be established. Analysis was conducted of pandemic prescribing in Israel compared with forecasted estimations of prescribing without the occurrence of the pandemic.^13^ This was found to demonstrate a significant association between increased prescribing incidence rates and the pandemic,^13^ with the data showing a 2% increase in actual prescribing compared with expectations without the pandemic.^13^ These differences in prescribing can be viewed alongside the changes that arose from the pandemic, for example, the effects of online consultations on the delivery and experience of primary care services, and how uncertainty of the development of the pandemic influenced decision-making and those healthcare staff making decisions.^14^

The present systematic review builds on initial global research into the incidence of antidepressant use during and following the pandemic. The aim was to improve insight into recent prescribing trends in the UK and Republic of Ireland. The geographic focus was chosen on the basis of pre-pandemic antidepressant prescribing data suggesting higher rates of prescribing in the UK compared with other European countries.^15^ Similarities between these countries include having publicly funded healthcare systems, experiencing similar effects of COVID-19, and overlap in governance and policies during the time of the pandemic (e.g. lockdown timings, restrictions and collaborative approaches taken).^16,17^ Therefore, these similarities and nuances provide opportunities for comparison, enriching the insights drawn while maintaining a cohesive scope for the study.

Specifically, we addressed the following research questions:

- What is the evidence for antidepressant prescribing rates for adults in the UK and Republic of Ireland during and post COVID-19?
- Did antidepressant drug prescriptions vary by drug, gender, location or age?

## Method

### Data search

Systematic searches were conducted using the APA PsycInfo, CINAHL, MEDLINE and Scopus databases. Searches were extended to include preprint databases medRxiv and Preprints.org. We utilised keywords related to antidepressants, prescribing, primary care, secondary care, tertiary care, adults and the UK (see Appendix A in the Supplementary Material, available at https://doi.org/10.1192/bjo.2026.10990, for the full search strategy). The portion of the search listing antidepressants was modelled on the search strategy created by Molenaar et al^18^ in their study of pregnancy and antidepressant use. This was modified to only include antidepressants licensed for use in the UK. The search was run on 5 November 2024 and was filtered to only include results published after 2020. The keywords ‘antidepressant’ and ‘prescribing’ were used.

### Eligibility criteria

The inclusion criteria specified the inclusion of observational studies that: (a) documented the number of prescribed antidepressants at two or more time points; (b) specified the recipients of the antidepressants to be adults (18 years old and above); (c) specified the recipients of prescriptions being sampled to be from the UK and/or the Republic of Ireland; (d) contained at least one time point after March 2020; and (e) were written in English. The review also permitted inclusion of conference papers and systematic reviews.

Specific antidepressant types included serotonin reuptake inhibitors (SSRIs), tricyclic and tricyclic-related antidepressants, serotonin–noradrenaline reuptake inhibitors (SNRIs) and monoamine oxidase inhibitors (MAOIs). These were the main categories of antidepressant, and specific antidepressants were limited to those licensed for use in the UK and Republic of Ireland.^19^ Prescriptions were considered to be eligible when prescribed by a primary, secondary or tertiary care facility and included initial, subsequent or repeat prescriptions from private or national healthcare providers. Studies were excluded if they assessed the efficacy of antidepressants, for example, in clinical trials or meta-analytic comparisons of effectiveness.

### Selection process

**Fig. 1:**
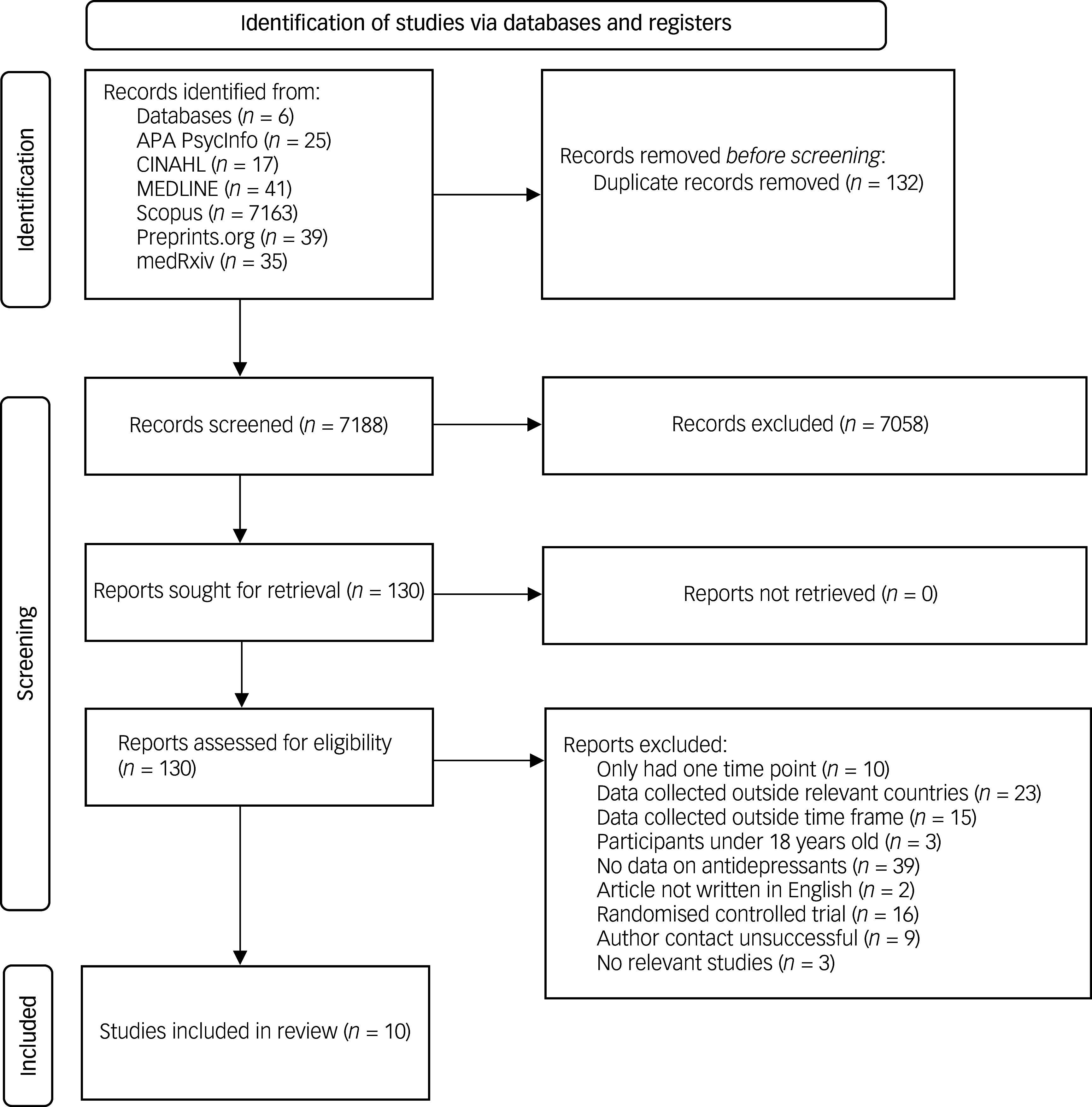
Preferred Reporting Items for Systematic reviews and Meta-Analyses flow diagram of included studies.

The selection process is illustrated in Fig. 1. The title and abstract screening and full-text screening were conducted by two researchers (M.J. and Mahmoud Elsherif) independently. Any discrepancies were discussed with the senior co-authors (E.M.K., S.J.T. and E.B.M.-L.) until a consensus was reached. The title and abstract screening had a percentage agreement of 98.4%, and the full-text screening had a percentage agreement of 93.9%. The data extraction process was conducted on 12 December 2024 by the lead author (M.J.) and on 9 March 2025 by a second reviewer (Mahmoud Elsherif). Following the same protocol as for screening, discrepancies were discussed with the other co-authors (E.M.K., S.J.T. and E.B.M.-L.). Authors of identified articles were contacted in instances in which data were unavailable. If no clarification was obtained, studies were removed from the review (see Appendix B in the Supplementary Material for excluded articles and authors contacted). Extracted data were retained as percentages or percentage changes where presented. Alternatively, when not presented as percentages (e.g. owing to different analysis methods used), raw data found in supplementary materials or provided by authors covering the study durations were used to calculate percentage changes for antidepressant prescribing. As a result, a final sample of ten studies was included in the review.

The Joanna Briggs Institute Prevalence Critical Appraisal Tool was used to detect bias in the studies included in this review.^20^ This was selected owing to its appropriateness for prevalence data and ratings of rigour compared with other tools.^21^ The articles were screened by two reviewers (M.J. and Mahmoud Elsherif) (see Appendix C in the Supplementary Material for tabulated risk of bias). Six studies met all nine of the nine criteria, and two studies achieved eight of nine, indicating good practice and lower risk of bias.^20^ One study was rated to meet 5–6 and another 7–8 of the nine criteria; however, following discussion with the secondary reviewers, these studies were included as they did not appear to be at high risk of bias that would compromise the study overall. The risk appraisal is summarised in Appendix C in the Supplementary Material.

### Data synthesis

The choice of analysis process was guided by the differing scopes and presentations of available data, which generated a heterogenous collection of findings that suggested meta-analysis would not be suitable. Therefore, the review utilised narrative synthesis to qualitatively compile and compare the findings. This was guided by the Preferred Reporting Items for Systematic reviews and Meta-Analyses SWiM (synthesis without meta-analysis) guidelines.^22^

The prescription amounts were displayed in an effect direction table that illustrated the direction of percentage change relative to the sample size.^23^ Although this method of data aggregation has been criticised for not recognising the strength of data types such as effect size or ratio, it was considered to be suitable for this review, as the prevalence data did not include effect sizes and were standardised by the percentage change specific to each sample in the study.

For the narrative synthesis, the data were initially grouped chronologically. This grouping enabled comparison of data by year across the timespans covered in the review and allowed the data to be placed within the context of when they were collected. The organisation and compilation of data in this way also differentiated the recency of trends in antidepressant prescribing. Other secondary variables collected were demographic characteristics, such as geographic location and participant age and gender. Secondary variable data were also grouped on the basis of study location and drug type reported to enable comparison and provide additional insight into any differences in prescription prevalence. Demographic characteristics were compared using descriptive statistics and discussed in the narrative synthesis.

### Changes from protocol

There were a few changes made from the original protocol. These were updated in the preregistration form. One change was the inclusion of the Republic of Ireland in the location criteria, as this broadened the scope of the review while being in geographical proximity to the other included countries. Likewise, the time frame specification was expanded to include studies that had comparisons of a time before the pandemic with a during or after pandemic time point, as opposed to both time points being required to be beyond the March 2020 cut-off.

## Results

The included studies reported antidepressant prescribing from 2004 to 2022. The study characteristics are summarised in Table 1.

**Table 1:** Characteristics of studies included

Author (year)	Study coverage	Sample size	Study type	Dates sampled	Data-set	JBI critical appraisal score^a^	Overall direction of change in prescribing
Armitage (2021)^24^	England	75 753 335	Retrospective analysis	2019 to 2020	NHS Digital	8/9	Increase
Booth et al (2021)^25^	Northern Ireland and England	159 937 072	Population-based data-linkage study	2019 to 2020	National Health Application and Infrastructure Services	9/9	Increase
Frazer and Frazer (2021)^26^	England, Wales, Scotland, Guernsey, Alderney, Jersey, Isle of Man	19 478 849	Retrospective analysis	March 2020 to May 2020	English Prescribing Dataset	8/9	Decrease
Williams et al (2020)^27^	Salford, England	449	Retrospective cohort study	March 2020 to May 2020	Salford Integrated Record	9/9	Decrease
Steeg et al (2022)^28^	UK	N/A	Retrospective cohort study	March to June 2010–19, 2020	UK Clinical Practice Research Datalink	7.5/9	Increase
Greene et al (2024)^29^	Scotland	472 648^b^	Cross-sectional analysis	2004 to 2021	Scottish Diabetes Research Network National Diabetes Study Dataset	9/9	Increase
Branford and Shankar (2022)^30^	England (individuals with intellectual disabilities)	N/A	Analysis	2020 to 2021	Clinical Practice Research Datalink; The Health Improvement Network	5.5/9	Increase
Naqvi et al (2022)^31^	London and Cornwall, England	210	Retrospective cohort study	January 2020 to January 2021	Clinical data from London and Cornwall	9/9	Increase and decrease^c^
Mattson et al (2024)^32^	Republic of Ireland	12 680 299	Interrupted time series	January 2016 to July 2022	Health Service Executive Primary Care Reimbursement service, General Medical Services scheme	9/9	Increase
Ruddock et al (2023)^33^	Northern Ireland	N/A	Review	April 2020 to March 2022	Northern Ireland Statistics and Research Agency	9/9	Increase and decrease^c^

NHS, National Health Service; N/A, not available.

a Scores were generated from the number of questions answered ‘Yes’, averaged from the scoring by two reviewers.

b For database studies (antidepressant amounts within database populations), the full database size was used.

c The study included separate groups that showed increases and decreases that could not be averaged.

### Antidepressant prescribing over time

The prescribing timespan covered in studies ranged from 3 months to 17 years. Of the ten included studies, nine reported on the prescribing of aggregated amounts of antidepressants. These studies combined different types of medication. Two studies provided a breakdown by individual drug. These studies documented changes in antidepressants prescribed across the period from 2004 to 2022. The percentage changes over time were compiled and tabulated (Table 2).

**Table 2:** Effect direction plot for numbers of items prescribed across the durations of the included studies

Study	Study design	2020	2021	2022	Change of antidepressants prescribed, %
Armitage (2021)^b^ ^24^	RA	▴			3.94
Booth et al (2021)^b^ ^25^	PDL		▴		5.44
Frazer and Frazer (2021)^b^ ^26^	RA	▾			−8.49
Williams et al (2020)^b^ ^27^	RCS	▾			−41.98
Steeg et al (2022)^a^ ^28^	RCS	▴			2.80
Greene et al (2024)^b^ ^29^	CSA		▴		43–73^c^
Branford and Shankar (2022)^a^ ^30^	RA		▴		3.40
Naqvi et al (2022)^b^ ^31^	RCS			•	−4.17 to +10.86^c^
Mattson et al (2024)^b^ ^32^	ITS			▴	30.42
Ruddock et al (2023)^a^ ^33^	Review		•	•	−3.10 to 1.90

RA, retrospective analysis; PDL, population data linkage study; RCS, retrospective cohort study; CSA, cross section analysis; ITS, interrupted time series.

Effect direction: upward arrow ▴, increase in antidepressant items, downward arrow ▾, decrease in antidepressant items, •, increase and decrease in antidepressant items.

Sample size: final item sample/cohort size: large arrow ▴ ≥ 75 753 335; medium arrow ▴, 450–75 753 334; small arrow ▴ ≤ 449.

The classification of small, medium or large studies is based on the cohort size/number of data items of which the median and upper and lower quartiles were calculated, therefore studies in the upper quartile were classified as larger comparative to those in the mid or lower quartiles.

Study quality, denoted by row colour: no colour, low risk of bias; shaded, some concerns (See Appendix C in the Supplementary Material).

a. Estimated database size.

b. Sample size.

c. The range of percentages across groups within this study as they were not aggregated.

As a result of variations in sampling among the studies, the direction and percentage of prescribing was considered with the cohort size in studies that used antidepressant prescription as a primary inclusion criterion and a prerequisite for involvement. However, in studies that looked at antidepressant prescribing as a secondary outcome or sampled the public overall, the percentage change reflected the number of data points relating to antidepressant prescribing used to represent the proportional cohort within the study. The use of cohort or sample measures are specified in the effect direction table (Table 2).

### Antidepressant prescribing by timespan

Seven of the studies used a pre-pandemic comparison sample.^24,28–30^ One of the pre-pandemic studies covered a cohort across the 3-month period of March to June from 2010 to 2019, with follow-up in the same 3-month period in 2020.^28^ The original cohort and comparison cohort comprised 48 739 and 4238 individuals, respectively.^28^ The cohorts in this study specifically consisted of participants who had a coded incident of self-harm and subsequent medication prescriptions. The prescribing incidences between March and June 2020 showed a 2.80% increase in prescribing compared with the previous data.^28^ A longer study covered the prescribing of antidepressants among individuals with type 2 diabetes in Scotland from 2004 to 2021, with a sample of 30 087 increasing in size to 95 736 by the later data collection point.^29^ The comparison between 2004 and 2021 data showed an average increase of 60% per 100 person-years in individuals sampled aged 55 years and older.^29^

Comparison with pre-pandemic prescribing was also done across smaller windows of time. Branford and Shankar^30^ captured antidepressant prescribing among individuals with intellectual disability; this comparison showed increases of 5% between 2010 and 2017 and 3.40% between 2017 and 2021. Therefore, all three studies that included pre-pandemic samples found an increase in antidepressant prescriptions. Mattson et al^32^ compared individual antidepressant drug types over a similar time, recording monthly prescriptions in the Republic of Ireland between January 2016 and July 2022. Likewise, Booth et al^25^ presented a summation of items prescribed in 2019 compared with 2020. Their analysis of more than 159 million prescription items across England and Northern Ireland showed a 5.44% increase in prescribing.^25^ Similarly, Armitage^24^ compared corresponding 5-month periods between April and September in both 2019 and 2020 and also found an increase of 3.94%.

Two studies, those by Williams et al^27^ and Frazer and Frazer,^26^ provided data on prescribing taking place between March and May 2020 in England and the UK. Both studies reported a decrease. Williams et al^27^ reported a decrease of 41.98%, based on a sample of 439 data points across the city of Salford, Greater Manchester. Frazer and Frazer^26^ conversely reported an 8.49% decrease in assessment of 19 478 849 antidepressant prescriptions across the UK.

The remaining two studies assessed later stages of the pandemic, comparing data from 2020 with data collected in 2022. Naqvi et al^31^ covered the period 2020 to 2021, reporting on prescribing for individuals with intellectual disability across a sample of 210 adults and two locations: rural Cornwall (*n* = 113) and urban London (*n* = 97). Differences between locations were noted, with the London sample showing the largest increase in dosage of antidepressants for patients with autism spectrum disorder (ASD; +10.86%), compared with a decrease of 0.48% for Cornwall. Data from Cornwall also showed a 4.17% decrease for individuals with intellectual disability. Ruddock et al^33^ investigated antidepressant prescribing in Northern Ireland using data collected between April 2020 and March 2021 and the equivalent months in 2021–2022. The percentage change in the Northern Irish population using antidepressants ranged from a decrease of 3.10% to an increase of 1.90%, depending on age and gender.^33^

### Antidepressant prescribing by drug

Four of the studies referred to separate drug types in their analysis of antidepressant prescriptions.^26,27,29,32^ Williams et al^27^ focused exclusively on SSRIs, whereas Frazer and Frazer^26^ looked at a large catalogue of different drugs, of which we extracted data for 26 antidepressants (six SSRIs, four MAOIs, two SNRIs, eight tricyclics, two tricyclic-related, and four others). Summation of these drugs showed an overall decrease in prescribed items across March to April 2020, with average decreases of −5.02% between March and April and −3.66% between April and May. The greatest decreases per individual drug among the SSRIs were for escitalopram (March to April, −8.04%) and fluvoxamine (April to May, −11.64%). The category of SSRIs showed the largest decrease between March and April (−6.78%), and MAOIs showed the largest decrease in the April to May data (−20.18%).

A smaller listing of antidepressants was used by Mattson et al,^32^ but the selection overlapped with those measured by Frazer and Frazer.^26^ Mattson et al^32^ also matched the findings of decreases across individual drug types between March and April 2020 (ranging from −2.81 to −6.07%). By contrast, with the exception of citalopram, they also found a rise in prescriptions between January and July 2020 and 2021. Between 2021 and 2022, prescriptions increased for amitriptyline (+5.87%), duloxetine (+3.10%), mirtazapine (+5.46%), sertraline (+7.66%) and venlafaxine (+3.27%), whereas they decreased for citalopram (−4.40%), escitalopram (−0.01%) and fluoxetine (−1.49%) in 2022. Greene et al^29^ also sampled the prescribing of antidepressants broadly and separated the prescribing of SSRIs, tricyclics and others (e.g. non-SSRI or non-tricyclic antidepressants such as SNRIs or MAOIs). Comparing prescribing between 2004 and 2021, SSRIs showed an average increase of 63.67%, compared with 34.67% for tricyclics and 183% for other antidepressants, among people in Scotland with type 2 diabetes.

### Antidepressant prescribing by location

Locations covered in the studies include single countries as well as combinations of countries within the UK and Republic of Ireland (Table 1). Three of the studies aggregated data across multiple countries,^25,26,28^ but the remaining studies focused on one country or distinguished data by country (England: 4; Northern Ireland: 1; Republic of Ireland: 1; Scotland: 1).^24,27,29,30–33^

Booth et al^25^ directly compared prescribing between England and Northern Ireland between 2019 and 2020, finding a larger relative increase in Northern Ireland, although this was not found to be statistically significant. Naqvi et al^31^ compared two locations within England (London and Cornwall) to draw a comparison between urban and rural prescribing. Their study demonstrated an average increase of 6.32% in the London cohort and an average decrease of 1.55% in Cornwall.

### Demographic differences

Some of the studies gave breakdowns by demographic variables (e.g. age, gender) or prerequisite characteristics or experiences (e.g. intellectual disability, recorded self-harm incidents). Ruddock et al^33^ provided data on the gender and age of individuals within their cohort. The cohort demonstrated a higher proportion of females administered antidepressants compared with males in both years sampled and across all age groups. In addition, antidepressant use was highest for the age group of 45–64 years and lowest for the group of 18–24 years. A similar finding was noted by Steeg et al,^28^ who reported that the modal group for receiving antidepressants comprised 25–64 -year-olds, compared with those 65 years old and older. Greene et al^29^ provided further insight into prescribing patterns in older adults with type 2 diabetes, reporting a 73% prescribing increase between 2004 and 2021 in those aged 55 to 64 years, a 64% increase in those aged 65–74 years, and a 43% increase in those aged 75 years and above.

Naqvi et al^31^ also sampled prescribing for adults with intellectual disability, including individuals with attention–deficit hyperactivity disorder (ADHD), ASD, or mild or moderate intellectual disability (as rated by the ICD-10).^34^ The percentage changes in antidepressant dosage in London and Cornwall, respectively, were +10.86 and −0.48% for patients with ASD, and −1.25 and 0% for patients with ADHD, whereas patients with intellectual disability only (without mental health comorbidity, i.e. ADHD or ASD) showed changes of +9.35 and −4.17%.

## Discussion

In this review, we aimed to understand antidepressant prescribing trends since the pandemic in the UK and Republic of Ireland. We used narrative synthesis to assess prescribing changes and differences in demographic factors such as age, gender and location. Most studies included in the review (*n* = 8) reported an increase in antidepressant prescriptions across the respective durations, with this increase ranging from 1.90 to 73%.^24,25,28–30,32,33^ However, two studies, by Frazer and Frazer^26^ and Williams et al,^27^ reported a decrease in prescribing, and another two studies (Naqvi et al^31^ and Ruddock et al^33^) reported both increases and decreases. Across these four studies, the decrease ranged from −3.10 to −41.99%.^26,27,31^ The two studies that reported only a decrease both relied on data from the period between March and May 2020.^26,30^ Differences in prescribing trends could be linked to specific time points during the pandemic.

In England, the early stages of the pandemic covered the implementation and legal enforcement of the first lockdown measures, an extension of the lockdown in April 2020 and conditional lifting of lockdown for those unable to work remotely.^35^ This time frame also involved a broader decrease in primary care access, for example, a 41% reduction in contact for children and young people in English primary care records during the first lockdown.^36^ It is therefore possible that the observed decreases in antidepressant prescribing between March and May 2020 reflected the political uncertainty during that time and were partly due to the associated hesitancy in accessing primary healthcare. The impact of the pandemic was also considered by Naqvi et al^31^ in their comparison of prescribing dosages between a rural and urban clinic during the pandemic. The study referenced differences in the environments and their potential power to affect antidepressant dosage. For example, the urban London environment, with typically less residential space, stricter lockdown enforcement, faster lifestyle pace and greater access to mental health services, may have contributed to a larger increase in antidepressant prescribing.

Our review also covered longer-term prescribing trends for more specific patient groups, for instance, a sizeable increase for individuals with type 2 diabetes across the 17 years of data collection up to 2021, from 43 to 73% across different age groups.^29^ The pattern of more modest yearly increases of around 3.94 to 5.44% align with past prescribing trends reported in England (+4.10% for 2016–17 to 2017–18 and +6.36% for 2018–19 to 2019–20).^37^ It could also be considered to align with other estimates of prescribing during the pandemic, for example, the comparison of prescribing of antidepressants across four healthcare insurance service records in Israel (+2% compared with estimates without the pandemic occurring).^13^ A study in north-east England (March 2018 to June 2023) analysed monthly antidepressant prescriptions, identifying seven phases of variation: prescriptions increased and then stabilised during the first lockdown and easing of restrictions, rose again with renewed restrictions, but declined during the second and third lockdowns; and post-lockdown, there was an initial rebound and a subsequent reduction. The results of that study, which was excluded from the present review owing to its inclusion of 17-year-olds, were consistent with some pre-pandemic trends but not with our findings of decreases during the first lockdown.^38^

Explorations of prescribing patterns for specific antidepressant types suggested a large decrease in prescriptions of SSRIs escitalopram and citalopram, as well as fluoxetine in later stages of the pandemic.^26,32^ Previous research has investigated the incidence of these drugs among prescribed antidepressants and the SSRI class in England between 2015 and 2019.^4^ Across the 4-year period preceding the pandemic, citalopram was the most prescribed antidepressant, with escitalopram also ranking in the top ten.^4^ By contrast, other studies reported increased prescribing trends for citalopram between 1998 and 2011, followed by a decrease between 2011 and 2018.^3^ This decrease in the second half of the 2010s matches the trend for citalopram found in the data within this review, which showed a decrease between 2020 and 2022.^3^ Prescribing trends for specific SSRIs need to be considered alongside relevant guidelines for antidepressant treatment. The National Institute for Health and Care Excellence guidelines do not encourage antidepressants as a first-line treatment, but, where appropriate, they do suggest SSRIs as a first-line option.^39^ Citalopram has been considered to be the recommended first-line treatment for depression in individuals with existing chronic pain.^39^ Hence, a decrease in prescribing could potentially be linked to factors such as incidence of first-time prescribing. Previous studies evaluating first-line prescribing also reported falling incidences for specific drugs. In data collected between 2009 and 2013, first-episode use fell by up to 1.4 and 1% for citalopram and fluoxetine, respectively, and recurrent use decreased by 3.3 and 1.8%.^40^ This may reflect prescribing changes due to decreased initiations of antidepressants during the pandemic; alternatively, it could be attributed to a continuation of trends in first-line prescribing that may have been established before the pandemic. It was impossible to resolve this question, because the data included in this review did not distinguish between first or repeat prescription of antidepressants. Future research could explore the role of first-line treatment choices and their impact on overall prescribing trends.

This review also reported differences in prescribing according to demographic characteristics such as age, gender and location, and among specific groups, such as individuals with intellectual disability or those with a history of self-harm. The data broadly highlighted females and middle-aged adults as having received more antidepressant prescriptions than males and other adult age groups. This was consistent with previous research, and the groups identified overlapped with some of the groups proposed to be at risk under the pressure of the pandemic, but not the risk groups of older adults who were especially vulnerable to the physical effects of COVID-19.^41^ A potential explanation is the use of wide age categories by several studies. For example, Steeg et al^28^ used categories with a spread of 40 years in their middle age category (24–64 years). Likewise, with the exception of Greene et al,^29^ who exclusively reported data from older age samples, all studies reviewed here included relatively few patients from the older age categories within their respective samples.

Nonetheless, the finding of females and middle-aged adults being most frequently prescribed to is also supported by data collected before the pandemic. A study assessing demographic variables associated with antidepressant use across a mental health service also found the proportion of females to be higher.^42^ The same study reported the mean age of those on one or more antidepressants to be 44.3 years.^42^ During the pandemic, the assessment of antidepressant prescribing in Israel also found higher proportions of women prescribed antidepressants compared with men.^13^ Research into the consumption of antidepressants across ages in Europe also suggested that the likelihood of antidepressant use peaked among people in their late 40s.^43^ This coincides with the modal groups receiving antidepressants found in this review. The higher prevalence of depression or anxiety disorders among women during the pandemic could have a plethora of explanations, for example, social and economic pressures such as familial or childcare responsibilities more frequently affecting women than men.^41^ In addition, potential differences in wages or financial security could account for different mental health needs between genders.^41^ These pressures could also have been exacerbated by further hardships arising from the pandemic, such as the cost-of-living crisis and continued strain on the National Health Service. This exacerbation may also have been felt by other groups that showed an increase in prescribing, such as individuals with intellectual disability or diabetes, or those living in rural or urban locations. A systematic review and meta-analysis by Farooqi et al^44^ proposed a higher prevalence of depression among individuals with diabetes compared with those without. In the context of the pandemic and diabetes, Steenblock et al^45^ noted the importance of recognising overlapping risks of depression and higher likelihood of developing severe COVID-19, on top of the general constraints of the pandemic (e.g. quarantine and lockdown isolation) and continuing to manage diabetes symptoms. Investigation of prescribing in individuals with intellectual disability has started, for example, through the development of the NHS STOMP (stopping over medication of people with a learning disability and autistic people) programme. This programme aims to raise awareness of the high risk and incidence of unnecessary prescribing within this group to discourage inappropriate prescribing.^8^

### Strengths and limitations

The review had the strength of being a comprehensive compilation of the data available on antidepressant prescribing in the UK and Republic of Ireland, synthesising comparisons made between past prescribing and that during the pandemic and in the present. Likewise, the review was preregistered in line with open science principles, and screening processes were conducted by multiple researchers to ensure validity.

A limitation was the ability to draw conclusions from a narrative synthesis. Although this method was chosen to suit the heterogeneity of the data collected in the review, it is inherently limited in comparison with other methods that analyse and compare the effect sizes of percentage changes. This could limit the conclusions of the study to only mapping and compiling findings across the pandemic, as opposed to making judgements regarding the significance of findings. The data sources used in the reviewed studies were often aggregated databases of clinical codes, which may have led to inclusion of antidepressant prescribing for symptoms of conditions other than mental health conditions (e.g. neuropathic pain), and not all studies provided a total sample size, which complicated comparisons. Clinical codes for antidepressant prescribing may differ from actual consumption by those receiving the prescriptions, but for the purposes of this review they were considered to provide acceptable insight into trends in the issuing and dispensing of antidepressants. In addition, some of the sampled populations included individuals with pre-existing conditions (e.g. diabetes and intellectual disability), which may have reduced the comparability of prescribing with that of the general population without these conditions. This was managed by acknowledging and reflecting on the respective populations of different studies and including studies owing to their contributions to antidepressant prescribing overall.

### Implications and future research

The present review evidenced a continued increase in antidepressant prescribing in the UK and the Republic of Ireland during and after the pandemic. Our results corroborate previous evidence of increased prescribing trends and may suggest increased demand for mental health support or rapid adaptations of healthcare systems to meet the unique challenges of the pandemic. However, the nature of this narrative synthesis did not allow an overall quantification of trends or indeed a statistical comparison with previous prescribing trends. Overall, our findings may raise concerns regarding the appropriateness of continued increases in prescribing and potential overtreatment, including consequences of avoidable side-effects and economic costs. However, further research is required to evaluate the appropriateness of current prescribing levels and identify psychosocial factors that may influence antidepressant treatment choices in prescribers, as this was beyond the scope of the present review.

The review highlighted a number of other areas that warrant further investigation, including the incidence of treatment initiation compared with repeat prescription, which could explain variations in prescribing trends for specific SSRIs recommended as first-line treatments. In addition, more in-depth investigation of specific patient groups, such as individuals with learning disabilities or those with diabetes, might provide deeper levels of insight into prescribing trends for the most vulnerable. Overall, more research is required to evaluate and contextualise antidepressant prescribing trends within wider concerns around overtreatment. Comparison of prescribing choices against National Institute for Health and Care Excellence guidelines and exploration of psychosocial factors that drive prescribing decisions could be helpful starting points.

## Supporting information

Jones et al. supplementary material Jones et al. supplementary material

## Data Availability

The data that support the findings are available from the corresponding author, M.J., upon reasonable request.

## Supplementary material

The supplementary material is available online at https://doi.org/10.1192/bjo.2026.10990
