# Supplementary material for: Antidepressant prescribing trends for adult patients in the UK and Ireland during the COVID-19 pandemic: systematic review
Source: BJPsych Open. 2026 Mar 2;12(2):e77. doi: 10.1192/bjo.2026.10990 (PMC12963840; doi:10.1192/bjo.2026.10990)
Supplement: Jones et al. supplementary material [file S2056472426109909sup001.docx]

**Appendix A**

**Search Strategy**

APA PsycINFO

(antidepressant OR "Serotonin Uptake Inhibitors" OR antidepress* OR anti-depress* OR ( serotonin* N3 ( uptake* OR reuptake* ) N3 inhibitor* ) OR ssri* OR ( depress* N3 ( drug OR agent* ) ) OR ( monoamin* N3 oxidas* N3 inhibitor* ) OR ( ( serotonin* OR noradrenalin* OR triple ) N3 ( reuptake OR uptake ) N3 inhibitor* ) OR tricyclic OR agomelatin* OR amitriptylin* OR citalopram* OR clomipramin* OR dosulepin* OR doxepin* OR duloxetin* OR escitalopram* OR fluoxetin* OR fluvoxamin* OR imipramin* OR isocarboxazid* OR lofepramin* OR lofepramin* OR mianserin* OR nortriptylin* OR phenelzin* OR paroxetin* OR reboxetin* OR sertralin* OR tranylcypromin* OR trazodon* OR trimipramine* OR venlafaxin* OR vortioxetin* ) AND ( "prescribing practice" OR prescrib* OR prescription ) AND ( "primary care" OR "primary medical care" OR "general practitioner" OR "general practice" OR "secondary care" OR "secondary medical care" OR "tertiary care" OR "tertiary medical care" ) AND ( adults OR aged OR "Middle Age" OR "Young Adult" ) AND ( UK OR "United Kingdom" OR England OR Scotland OR Wales OR "Northern Ireland")

CINAHL

( antidepressant OR "Serotonin Uptake Inhibitors" OR antidepress* OR anti-depress* OR ( serotonin* N3 ( uptake* OR reuptake* ) N3 inhibitor* ) OR ssri* OR ( depress* N3 ( drug OR agent* ) ) OR ( monoamin* N3 oxidas* N3 inhibitor* ) OR ( ( serotonin* OR noradrenalin* OR triple ) N3 ( reuptake OR uptake ) N3 inhibitor* ) OR tricyclic OR agomelatin* OR amitriptylin* OR citalopram* OR clomipramin* OR dosulepin* OR doxepin* OR duloxetin* OR escitalopram* OR fluoxetin* OR fluvoxamin* OR imipramin* OR isocarboxazid* OR lofepramin* OR lofepramin* OR mianserin* OR nortriptylin* OR phenelzin* OR paroxetin* OR reboxetin* OR sertralin* OR tranylcypromin* OR trazodon* OR trimipramine* OR venlafaxin* OR vortioxetin* ) AND ( "prescribing practice" OR prescrib* OR prescription ) AND ( "primary care" OR "primary medical care" OR "general practitioner" OR "general practice" OR "secondary care" OR "secondary medical care" OR "tertiary care" OR "tertiary medical care" ) AND ( adults OR aged OR "Middle Age" OR "Young Adult" ) AND ( UK OR "United Kingdom" OR England OR Scotland OR Wales OR "Northern Ireland")

MEDLINE

(exp antidepressant/ or Serotonin Uptake Inhibitors/ or (antidepress* or anti-depress* or (serotonin* adj3 (uptake* or reuptake*) adj3 inhibitor*) or SSRI* or sertralin* or paroxetin* or fluvoxamin* or fluoxetin* or citalopram* or escitalopram* or (depress* adj3 drug) or (monoamin* adj3 oxidas* adj3 inhibitor*) or ((serotonin* or noradrenalin* or triple) adj3 (reuptake or uptake) adj3 inhibitor*) or tricyclic or agomelatin* or amitriptylin* or citalopram* or clomipramin* or dosulepin* or doxepin* or duloxetin* or escitalopram* or fluoxetin* or fluvoxamin* or imipramin* or isocarboxazid* or lofepramin* or lofepramin* or mianserin* or nortriptylin* or phenelzin* or paroxetin* or reboxetin* or sertralin* or tranylcypromin* or trazodon* or trimipramine* or venlafaxin* or vortioxetin*).mp.) and (prescrib*.mp. or exp prescription/) and (primary care.mp. or exp general practitioner/ or general practi*.mp. or exp general practice/ or family practi*.mp. or secondary care.mp. or tertiary care.mp.) and (Adults or Aged or Middle Age or Young Adult).mp. and (UK.mp. or exp United Kingdom/ or exp England/ or exp Scotland/ or exp Wales/ or exp Northern Ireland/)

Scopus

( antidepressant OR "Serotonin Uptake Inhibitors" OR antidepress* OR anti-depress* OR ( serotonin* W/3 ( uptake* OR reuptake* ) W/3 inhibitor* ) OR ssri* OR ( depress* W/3 ( drug OR agent* ) ) OR ( monoamin* W/3 oxidas* W/3 inhibitor* ) OR ( ( serotonin* OR noradrenalin* OR triple ) W/3 ( reuptake OR uptake ) W/3 inhibitor* ) OR tricyclic OR agomelatin* OR amitriptylin* OR citalopram* OR clomipramin* OR dosulepin* OR doxepin* OR duloxetin* OR escitalopram* OR fluoxetin* OR fluvoxamin* OR imipramin* OR isocarboxazid* OR lofepramin* OR lofepramin* OR mianserin* OR nortriptylin* OR phenelzin* OR paroxetin* OR reboxetin* OR sertralin* OR tranylcypromin* OR trazodon* OR trimipramine* OR venlafaxin* OR vortioxetin* ) AND ( "prescribing practice" OR prescrib* OR prescription ) AND ( "primary care" OR "primary medical care" OR "general practitioner" OR "general practice" OR "secondary care" OR "secondary medical care" OR "tertiary care" OR "tertiary medical care" ) AND ( adults OR aged OR "Middle Age" OR "Young Adult" ) AND ( UK OR "United Kingdom" OR England OR Scotland OR Wales OR "Northern Ireland")

Preprint Databases

Medrxiv

antidepressant AND prescribing AND (UK or 'United Kingdom' or England or 'Northern Ireland' or Wales or Scotland) AND adult

Preprints.org

Antidepressant prescribing

**Appendix B**

**Excluded Articles**

Table 1B. A Table of Full-text Article Exclusion

| **Author** | **Reason for removal MJ** | **Reason for removal ME** |
| --- | --- | --- |
| Aggarwal (2024) | No data on antidepressants amount | No data on antidepressants amount |
| Aguiar et al  (2022) | No data on antidepressants amount | No data on antidepressants amount |
| Alfageh et al (2020) | Data collected outside of timeframe | Data collected outside of timeframe |
| Alhlayl, et al  (2022) | Article not written in English | Data collected outside of timeframe |
| Allary et al (2024) | Data collected outside relevant countries | Data collected outside relevant countries |
| Alsugeir et al (2024) | No data on antidepressants amount | No data on antidepressants amount |
| Armitage (2021) | No data on antidepressants amount | No data on antidepressants amount |
| Badamasi et al (2021) | No data on antidepressants amount | Data collected outside of timeframe |
| Ball et al (2023) | Participants under 18 | Only have one timepoint |
| Bansal et al (2022) | Only have one timepoint | Data collected outside of timeframe |
| Barker et al (2024) | Data collected outside relevant countries | Data collected outside relevant countries |
| Barnes et al (2024) | Article not written in English | Only have one timepoint |
| Blackwell et al (2022) | Data collected outside of timeframe | Data collected outside of timeframe |
| Bogowicz et al (2021) | Data collected outside of timeframe | Data collected outside of timeframe |
| Brender et al (2021) | No data on antidepressants amount | No data on antidepressants amount |
| Bu et al (2021) | Only have one timepoint | No data on antidepressants amount |
| Capuzzi et al (2021) | Data collected outside relevant countries | Data collected outside relevant countries |
| Chen et al (2023) | No data on antidepressants amount | No data on antidepressants amount |
| Christensen et al (2023) | Data collected outside relevant countries | Data collected outside relevant countries |
| Costello et al (2023) | No data on antidepressants amount | No data on antidepressants amount |
| Crowe et al (2023) | No data on antidepressants amount | Data collected outside relevant countries |
| De Crescenzo et al (2020) | RCT | RCT |
| Denee et al (2021) | Data collected outside of timeframe | Data collected outside of timeframe |
| Diaz-Camal et al (2022) | No data on antidepressants amount | No data on antidepressants amount |
| Ding et al (2022) | No data on antidepressants amount | Participants under 18 |
| Duffy et al (2024) | No data on antidepressants amount | No data on antidepressants amount |
| Dugosh et al (2023) | Only have one timepoint | No data on antidepressants amount |
| Eleftheriadou et al (2024) | Data collected outside of timeframe | Contact Author |
| Eller et al (2023) | No data on antidepressants amount | Data collected outside relevant countries |
| Engelmann et al (2021) | No data on antidepressants amount | Data collected outside relevant countries |
| Erritzoe et al (2024) | RCT | RCT |
| Fabbri et al (2021) | Data collected outside of timeframe | Contact Author |
| Francia et al (2024) | No relevant studies | Data collected outside of timeframe |
| Fu et al (2024) | Participants under 18 | Contact Author |
| García-Marín et al (2022) | No data on antidepressants amount | No data on antidepressants amount |
| Garel et al (2023) | Data collected outside relevant countries | Data collected outside relevant countries |
| Garel et al (2023) | No data on antidepressants amount | No data on antidepressants amount |
| Gerritsen et al (2021) | Data collected outside of timeframe | Data collected outside relevant countries |
| Gougoulaki et al (2021) | RCT | RCT |
| Greene et al (2023) | No relevant studies | Only have one timepoint |
| Håkansson et al (2022) | Data collected outside relevant countries | Data collected outside relevant countries |
| Hamlin et al (2023) | Data collected outside relevant countries | Data collected outside relevant countries |
| Hang et al (2021) | RCT | Only have one timepoint |
| Hayes et al (2024) | No data on antidepressants amount | No data on antidepressants amount |
| Heald et al (2020) | Data collected outside of timeframe | Data collected outside of timeframe |
| Henking et al (2023) | No data on antidepressants amount | Contact Author |
| Horowitz et al (2022) | No data on antidepressants amount | No data on antidepressants amount |
| Horowitz (2023) | No data on antidepressants amount | No data on antidepressants amount |
| Izza et al (2020) | Data collected outside of timeframe | Data collected outside of timeframe |
| Jaramillo et al (2023) | No data on antidepressants amount | No data on antidepressants amount |
| Jung et al (2020) | RCT | Data collected outside of timeframe |
| Kamp et al (2024) | RCT | Data collected outside relevant countries |
| Kato et al (2023) | RCT | Data collected outside relevant countries |
| Kazdin et al (2024) | Only have one timepoint | Data collected outside relevant countries |
| Kelly et al (2021) | No data on antidepressants amount | Participants under 18 |
| Kendrick et al (2024) | RCT | RCT |
| Khawagi et al (2022) | Data collected outside of timeframe | Data collected outside of timeframe |
| Khorassani et al (2024) | RCT | RCT |
| Kiecka et al (2022) | No data on antidepressants amount | No data on antidepressants amount |
| Kim et al (2021) | No data on antidepressants amount | No data on antidepressants amount |
| Koo et al (2023) | Data collected outside relevant countries | Data collected outside of timeframe |
| Kunzler et al (2023) | No relevant studies | Data collected outside of timeframe |
| Larkin et al (2022) | No data on antidepressants amount | No data on antidepressants amount |
| Lee et al (2023) | No data on antidepressants amount | No data on antidepressants amount |
| Liao et al (2023) | Only have one timepoint | Only have one timepoint |
| Liberman et al (2022) | Data collected outside relevant countries | No data on antidepressants amount |
| Lim et al (2020) | RCT | No data on antidepressants amount |
| Lin et al (2020) | Data collected outside relevant countries | Data collected outside relevant countries |
| Lovegrove et al (2024) | No data on antidepressants amount | Only have one timepoint |
| Ma et al (2023) | Only have one timepoint | Data collected outside of timeframe |
| Macdonald et al (2023) | No data on antidepressants amount | No data on antidepressants amount |
| Mahesarajah et al (2024) | Data collected outside relevant countries | Contact Author |
| Mahmood et al (2024) | Only have one timepoint | Only have one timepoint |
| Malamud et al (2024) | RCT | RCT |
| Marconi et al (2023) | Data collected outside relevant countries | Data collected outside relevant countries |
| Martella et al (2024) | Data collected outside relevant countries | Data collected outside relevant countries |
| Mas et al (2022) | Data collected outside relevant countries | No data on antidepressants amount |
| Meißner et al (2024) | Only have one timepoint | Data collected outside relevant countries |
| Mohan et al (2024) | Data collected outside relevant countries | Data collected outside relevant countries |
| Montano et al (2023) | No data on antidepressants amount | No data on antidepressants amount |
| Murphy et al (2022) | Only have one timepoint | Only have one timepoint |
| Ng et al (2024) | Data collected outside of timeframe | Data collected outside relevant countries |
| Nijs et al (2020) | RCT | Data collected outside relevant countries |
| Norman (2021) | No data on antidepressants amount | No data on antidepressants amount |
| O'Neill et al (2021) | Data collected outside of timeframe | Data collected outside of timeframe |
| Paton et al (2020) | Data collected outside of timeframe | Data collected outside of timeframe |
| Plasencia-García et al (2022) | RCT | RCT |
| Powell et al (2021) | Data collected outside relevant countries | Data collected outside relevant countries |
| Rahman et al (2024) | Only have one timepoint | RCT |
| Resnick et al (2024) | Data collected outside relevant countries | Data collected outside relevant countries |
| Samel et al (2022) | No data on antidepressants amount | No data on antidepressants amount |
| Sanchez-Ruiz et al (2023) | Data collected outside relevant countries | Data collected outside relevant countries |
| Siraj et al (2022) | Data collected outside of timeframe | No data on antidepressants amount |
| Sjaarda et al (2020) | Data collected outside relevant countries | Data collected outside relevant countries |
| Solis (2020) | No data on antidepressants amount | No data on antidepressants amount |
| Solmi et al (2021) | No data on antidepressants amount | No data on antidepressants amount |
| Soyemi et al (2022) | Data collected outside relevant countries | Data collected outside relevant countries |
| ter Hark et al (2022) | RCT | Data collected outside relevant countries |
| Tiger et al (2024) | Data collected outside relevant countries | Data collected outside relevant countries |
| Van Haaren et al (2023) | Data collected outside of timeframe | Data collected outside of timeframe |
| Van Leeuwen et al (2020) | No data on antidepressants amount | Data collected outside relevant countries |
| Vas et al (2023) | No data on antidepressants amount | Data collected outside relevant countries |
| Venkataraman et al (2023) | Data collected outside relevant countries | Data collected outside relevant countries |
| Walker et al (2021) | No measure of antidepressants | No data on antidepressants amount |
| Wang et al (2024) | Participants under 18 | No data on antidepressants amount |
| Wu et al (2023) | RCT | RCT |
| Yi et al (2022) | No data on antidepressants amount | Data collected outside relevant countries |
| Zhang et al (2022) | Data collected outside relevant countries | Data collected outside relevant countries |
| Zhang et al (2024) | Data collected outside relevant countries | Data collected outside relevant countries |
| Zhao et al (2023) | RCT | Data collected outside relevant countries |
| Zhao et al (2024) | No data on antidepressants amount | No data on antidepressants amount |
| Zwiebel & Viguera (2022) | No data on antidepressants amount | No data on antidepressants amount |

LEGEND

Study’s organised alphabetically

Studies recorded under first breach of systematic review inclusion criteria

Table 2B. A Table of Authors Contacted

| **AUTHOR CONTACTING** |  |
| --- | --- |
| Carr, M. J., et al. (2021) | Author contacting unsuccessful |
| Curtis, H. J., et al. (2023) | Author contacting unsuccessful |
| Dykxhoorn, J., et al (2024). | Author contacting unsuccessful |
| Maguire, A., et al. (2022) | Author contacting unsuccessful |
| McCool, A., et al. (2022) | Author contacting unsuccessful |
| Pappa, S., et al. (2024) | Author contacting unsuccessful |
| Rabeea, S. A., et al. (2021). | Author contacting unsuccessful |
| Taxiarchi, V. P., et al. (2023) | Author contacting unsuccessful |
| Wang, Y., et al. (2023). | Author contacting unsuccessful |

LEGEND

Study’s organised alphabetically

**Appendix C**

**Table of Risk of Bias Ratings**

| Paper | Reviewer | Was the sample frame appropriate to address the target population? | Were study participants sampled in an appropriate way? | Was the sample size adequate? | Were the study subjects and the setting described in detail? | Was the data analysis conducted with sufficient coverage of the identified sample? | Were valid methods used for the identification of the condition? | Was the condition measured in a standard, reliable way for all participants? | Was there appropriate statistical analysis? | Was the response rate adequate, and if not, was the low response rate managed appropriately? | Total |
| --- | --- | --- | --- | --- | --- | --- | --- | --- | --- | --- | --- |
| Armitage (2021) | MJ | Yes    Looking at services in England so utilized nhs data | Yes    Used all data available from records | Yes    No full total given of people within database – but presents million+ items | No    No demographic data given, only specified as adults | Yes    England national sample | Yes    Clinical data codes from database | Yes    All records from primary care | Yes    Basic descriptive stats (percentage change) and all numbers reported | Yes    Unspecified amount but very large sample | 8/9 |
|  | ME | Yes | Yes | Yes | Yes | No | Yes | Yes | Yes | Yes | 8/9 |
| Booth et al (2021) | MJ | Yes  Aiming for 2 populations so sampled 2 national databases | Yes  All available from databases | Yes  Multiple million datapoints | Yes  Specified by country but not individual characteristics | Yes  Large Cohort | Yes  Analysed diagnostic/database codes | Yes  Objective measure from records | Yes  Descriptive statistics and T-Tests | Yes | 9/9 |
|  | ME | Yes | Yes | Yes | Yes | Yes | Yes | Yes | Yes | Yes | 9/9 |
| Frazer and Frazer (2021) | MJ | Yes    Not show demographics but use all prescriptions in primary care so theoretically includes all | Yes    Used all data available | Yes    National level dataset so power calculations | No    The locations were listed but no demographic data, but locations prove population of interest | Yes    Analyzed whole sample | Yes    Analyzed using diagnostic labels – some room for error e.g misclassification, not specified as depression only prescribing | Yes    Objective measure | Yes    Summed actual items compared to forecasting | Yes    National Sample | 8/9 |
|  | ME | Yes | Yes | Yes | No | Yes | Yes | Yes | Yes | Yes | 8/9 |
| Williams et al (2020) |  | Yes    National dataset in Salford | Yes    Used all data available | Yes    Aggregated data across location | Yes    Some demographic data – gender, age | Yes    Analyzed whole sample | Yes  Analyzed using diagnostic labels – some room for error e.g misclassification, not specified as depression only prescribing | Yes    Objective measure | Yes    Model of expected compared to observed and plotted, compared to confidence intervals, plotted and tabulated | Yes    Area sample | 9/9 |
|  | ME | Yes | Yes | Yes | Yes | Yes | Yes | Yes | Yes | Yes | 9/9 |
| Steeg et al (2021) | MJ | Yes    Wanted UK so have databases from each country | Yes    All available from databases | Yes    Nearly 50k participants | No    No demographic info shared | Yes    Used all available | Yes    Analysed diagnostic codes | Yes    Objective measure from records | Yes    Modelling for the observed and actual comparison- data and CI reported | Yes | 8/9 |
|  | ME | Yes | Yes | Yes | No | Yes | Yes | Yes | Yes | No | 7/9 |
| Greene et al (2024) | MJ | Yes  99% coverage of primary and secondary care data for people with diabetes | Yes    All available from databases | Yes  High coverage of all eligible participants | Yes  Demographic information available | Yes  Used all available | Yes    Analysed diagnostic codes | Yes    Objective measure from records | Yes  Prevalence calculations | Yes | 9/9 |
|  | ME | Yes | Yes | Yes | Yes | Yes | Yes | Yes | Yes | Yes | 9/9 |
| Branford and Shankar (2022) | MJ | Yes    Adults across all NHS regions – not everyone but large proportion | Yes    Used all data from records | Unsure    No clear total of participants given in paper | No    No demographics given only age being above 18 and type of drug | Yes    Analysed whole sample | Yes    Analyzed using diagnostic labels in database | Yes    All from GP records | Yes    Descriptive statistics on incidence rising, includes CIs, reported in text and graphically | Unsure    Drawn from national samples but unsure of how many | 6/9 |
|  | ME | Yes | Yes | Yes | No | No | Yes | Yes | No | No | 5/9 |
| Naqvi et al (2022) | MJ | Yes  Sampled mental health service users with intellectual disability, sampled across two clinics | Yes  All eligible records across two clinics | Yes  *N* = 210, populations across two clinics, but different male to female ratio | Yes  Demographic data provided | Yes  All participants had an intellectual disability as part of inclusion | Yes  All data drawn from clinical codes | Yes  All recorded using same codes | Yes  Chi-Squared and percentage change | Yes  All eligible service users across two clinics | 9/9 |
|  | ME | Yes | Yes | Yes | Yes | Yes | Yes | Yes | Yes | Yes | 9/9 |
| Mattson et al (2024) | MJ | Yes    32% of the population so large proportion  Means tested healthcare group | Yes    All available from database | Yes    62 million prescriptions | Yes    Lots of demographic data – gender, age comorbidities | Yes    Analyzed whole sample | Yes    Analysed diagnostic codes – but not specific to just depression | Yes    Objective measure | Yes    Calculated forecasting intervals, and plotted dispensing, plots and CIs reported | Yes    National Sample | 9/9 |
|  | ME | Yes | Yes | Yes | Yes | Yes | Yes | Yes | Yes | Yes | 9/9 |
| Ruddock et al (2023) | MJ | Yes  Using a national statistics report for Republic of Ireland | Yes  Used nation wide prescribing data | Yes  Covered the whole country | Yes  Lots of demographic data | Yes  Used all data available of whole country | Yes  Analysed prescription data | Yes  All drawn from medical records | Yes  Graphical comparison over time | Yes  Country wide coverage | 9/9 |
|  | ME | Yes | Yes | Yes | Yes | Yes | Yes | Yes | Yes | Yes | 9/9 |
